# Weekly hypofractionated radiation therapy in elderly non-resectable cutaneous squamous cell carcinoma of the head and neck region

**DOI:** 10.1007/s11547-020-01260-5

**Published:** 2020-10-15

**Authors:** Francesca De Felice, Daniela Musio, Vincenzo Tombolini

**Affiliations:** Department of Radiotherapy, Policlinico Umberto I “Sapienza” University of Rome, Viale Regina Elena 326, 00161 Rome, Italy

**Keywords:** Head neck cancer, Cutaneous squamous cell carcinoma, Oral cavity, Radiotherapy, Hypofractionation, Elderly

## Abstract

**Introduction:**

Treatment of inoperable cutaneous squamous cell carcinoma (cSCC) of the head and neck region is still debated.

**Case report:**

We report an original case of cure of cSCC of the head and neck region with weekly hypofractionated radiation therapy with megavoltage electrons prescribed for locally advanced inoperable disease.

**Results:**

Weekly hypofractionated radiotherapy assured complete regression and was well-tolerated.

**Conclusion:**

The real efficacy of this treatment in the therapeutic arsenal remains to be defined. A clinical trial is ongoing to test the use of 8 weekly fractions of 8 Gy hypofractionated RT regimens in non-resectable cSCC cases.

## Introduction

Cutaneous squamous cell carcinoma (cSCC) represents the second most frequent form of non-melanoma skin cancer, with a rising incidence in elderly population (year by year more than 7%) [1]. But its estimated cases for 2020 cannot be predicted because incidence data are not collected by most cancer registries worldwide [2]. cSCC predominantly derives from the malignant proliferation of epidermal keratinocytes. While complete surgical resection is the main standard approach, when feasible, at present, the level of evidence of treatment options for non-resectable cSCC cases is low [3]. We report for the first time a complete response in a elderly patient with a non-resectable cSCC of the cheek treated with weekly hypofractionated radiation therapy with megavoltage electrons. The aim is to share our treatment approach and provide some directions for future research in the management of non-resectable cSCC patients.

## Case description

**Fig. 1:**
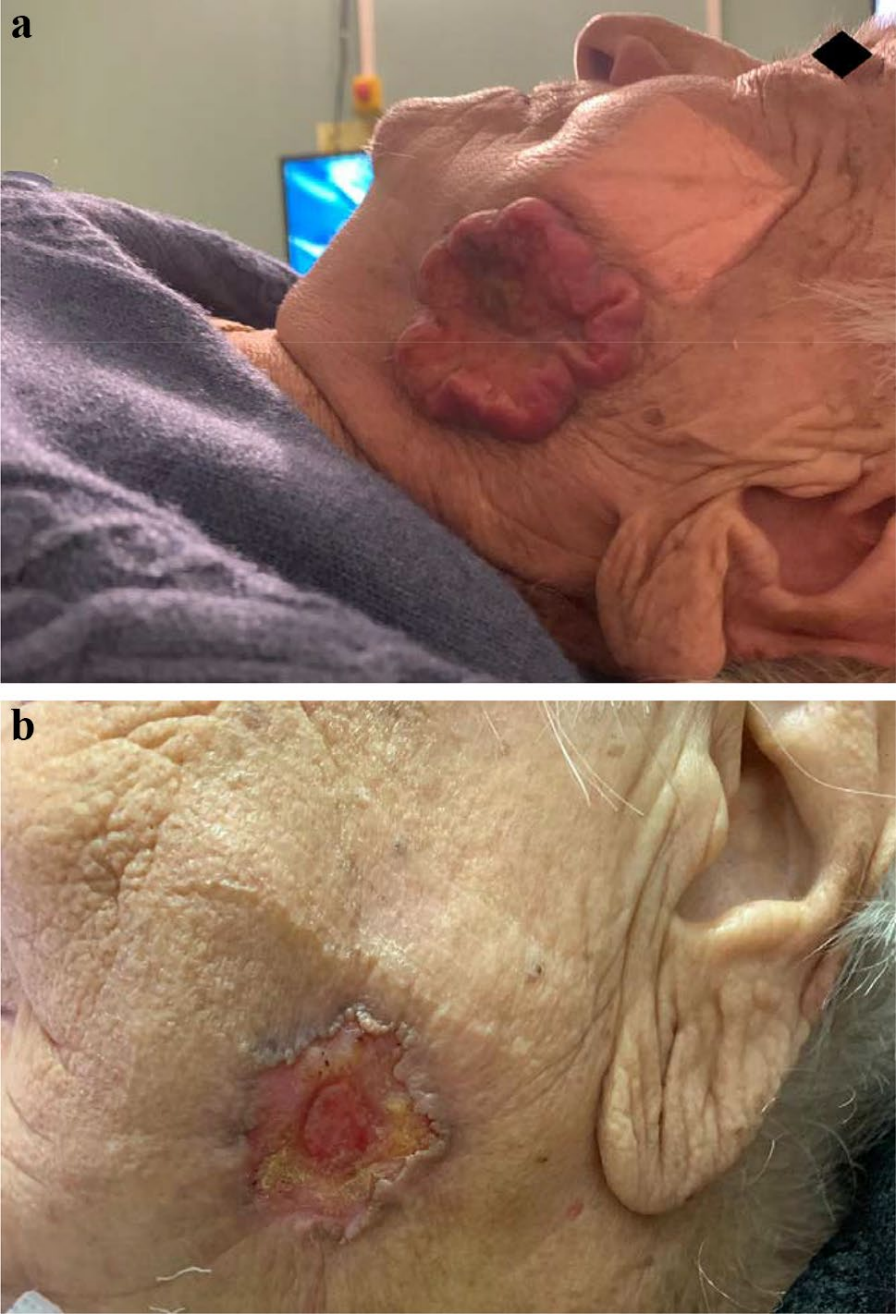
Cutaneous squamous cell carcinoma at diagnosis (**a**) and after treatment (**b**)—8 weekly fractions of 8 Gy hypofractionated radiation therapy with megavoltage electrons

In October 2019, a 94-year-old woman, with difficulties in mobilizing, cardiovascular comorbidities and cognitive impairment, consulted for a singular large inoperable cSCC on the left cheek, measuring 4.5 cm × 4.5 cm in size (cT3 cN0 cMx) (Fig. 1). Biopsy confirmed a well-differentiated cSCC. Radiological assessment revealed no loco-regional lymph node involvement, and there were no distant metastasis. Treatment with radiotherapy was decided at a multidisciplinary team meeting. Based on tumor characteristics—circular shape, indented margins, 25 mm thickness and anatomic location—and surrounding normal tissue considerations, the physical properties of MeV electron therapy—rapid dose falloff sparing deeper structures, such as oral cavity and mandible bone—were preferred. A total dose of 64 Gy in 8 weekly fractions of 8 Gy was prescribed—biological equivalent dose (BED) equal to 92.1 Gy with α/β = 10 for early responding tissues [to note we completed the calculations for tumor BED, using the seventh LQ formula allowing for cell proliferation [4]: BED = [total dose × RE] minus [ln2(T–Tk)/αTp]]. A single appositional field using an electron energy of 9 MeV for 6 × 6 applicator was chosen so that the lesion was encompassed by the 95% of dose at the deep margin. Bolus material was not applied to reduce inhomogeneous dose distributions, because of the high risk of air gap formation. This field arrangement permitted to adequately cover the basal extent of the tumor lesion. Radiation oncologist performed weekly visual confirmation of surface coverage before treatment. The patient well tolerated treatment with expected effects on lesion regression (Fig. 1). No skin reaction was present in the shielded regions on the face.

## Discussion

Our weekly hypofractionated radiation therapy with megavoltage electrons scheme assured complete cSCC removal and maximal preservation of function and cosmesis. Treatment compliance was high. Interestingly, our hypothesis to cover the deepest level of tumor invasion without achieving dose uniformity in the overall lesion resulted in an optimal clinical response. From a technical point of view, electrons are particularly important in the treatment of superficial lesions. Definition of treatment field is complicated in cSCC with high borders, making it difficult to identify the optimal electron driver energy. In this context, we have chosen to prioritize the dose to deeper tumor layers resulting in cold spot beneath the proximal surface of the lesion margins and hot spot in the distal surface. This confirms the concept that there is a high cell turnover, continuously renewing skin containing rapidly proliferating and maturing cells. Therefore, radiation-related damage to the basal keratinocytes interferes with malignant cells proliferation, on one hand, and normal epithelial cells production and maturation, on the other hand. Weekly hypofractionated dose improves the balance between tumor control and skin tissue repair. Moreover, the choice of hypofractionated regimen using electron beam therapy without three-dimensional planning is considered more cost-effective in terms of financial, human and physical resources [5]. As clinicians involved in cancer patient care, we believe that a patient-centred management is a major goal, but it should be reached by high-quality treatment and evidence-based research.

In elderly, adequate life quality, both for patient and his/her family, is a critical and inseparable component of comprehensive cSCC treatment. Weekly hypofractionated approach can reduce problems with transportation, without compromise clinical outcome.

Just now, the American Society for Radiation Oncology (ASTRO) summarized the wide variety of appropriate regimens able to provide excellent local control with good cosmetic outcomes in cSCC [6]. The suggested hypofractionation schedules for primary site management are listed in Table 1. Although the strength of recommendation is strong, the quality of evidence is low.

**Table 1:** American Society for Radiation Oncology (ASTRO) hypofractionation schemes [5]

Total dose (Gy)	Dose per fraction (Gy)	Number of fraction	Weekly fraction
40	5	8	2
40.5	4.5	9	Twice daily for 9 fraction/week
44	4.4	10	4
44	3 (first dose) 4 (last dose)	14	Twice daily for 10 fraction/week
45	5	9	Twice daily for 9 fraction/week
45	4.5	10	4
45	3	15	5
48	3	16	5
50	2.5	20	5
51	3	17	5
54	3	18	4–5
55	2.75	20	5
61.2	3.4	18	5

With this in mind, if proved safe, effective and reliable, the 8 weekly fractions of 8 Gy hypofractionated regimen has the potential to become a standard approach for elderly cSCC patients management. In order to standardize the proposed radiation fractionation scheme, a clinical trial for use of 8 weekly fractions of 8 Gy hypofractionated radiotherapy with megavoltage electrons in the elderly cSCC setting is ongoing.

## Conclusion

Elderly cSCC patients can benefit from hypofractionated radiotherapy with megavoltage electrons leading to control the cancer in the most effective way possible and to maximally preserve their autonomy and/or family assistance. Results from the ongoing clinical trial of this approach will inform how we can best treat in future elderly patients with advanced cSCC.
